# The role of bone‐modifying agents in myeloma bone disease

**DOI:** 10.1002/jbm4.10518

**Published:** 2021-06-15

**Authors:** Huifang Lu, Xerxes Pundole, Hans C. Lee

**Affiliations:** ^1^ Department of General Internal Medicine Section of Rheumatology and Clinical Immunology Houston Texas USA; ^2^ Department of Health Services Research The University of Texas MD Anderson Cancer Center Houston Texas USA; ^3^ Department of Lymphoma/Myeloma The University of Texas MD Anderson Cancer Center Houston Texas USA; ^4^ Present address: Amgen Inc. Thousand Oaks CA USA

**Keywords:** ANABOLICS, ANTIRESORPTIVES, CANCER, OSTEOBLASTS, OSTEOCLASTS

## Abstract

Bone disease is common in patients with multiple myeloma (MM), which manifests as bone pain and skeletal‐related events (SREs) such as pathological fractures and spinal cord compression. Myeloma bone disease (MBD) can adversely affect the quality of life of patients and have negative effects on morbidity and mortality. The pathogenesis of MBD is complex, and several factors are involved in the dysregulation of bone metabolism and uncoupling of bone remodeling, which result in net bone loss and devastating SREs. Broadly speaking, elevated osteoclast activity, suppressed osteoblast activity, and an aberrant marrow microenvironment play a role in MBD. Interaction of MM cells with the main bone cell osteocytes also promote further bone destruction. This review focuses on the role of bone‐modifying agents in the prevention and treatment of MBD. The mainstay of MBD prevention are antiresorptive agents, bisphosphonates and denosumab. However, these agents do not play a direct role in bone formation and repair of existing MBD. Newer agents with anabolic effects such as anti‐sclerostin antibodies, parathyroid hormone, anti‐Dickkopf‐1 antibodies, and others have shown potential in repair of MBD lesions. With the development of several new agents, the treatment landscape of MBD is likely to evolve in the coming years. © 2021 The Authors. *JBMR Plus* published by Wiley Periodicals LLC on behalf of American Society for Bone and Mineral Research.

## Overview and Epidemiology

### Multiple myeloma

Multiple myeloma (MM) is a neoplasm caused by malignant proliferation of plasma cells in the bone marrow. It is characterized by the production of monoclonal immunoglobulins, which can lead to end organ damage. MM presents commonly as anemia, bone pain (with skeletal lesions), hypercalcemia, and kidney failure.^(^ ^1^, ^2^ ^)^ It is diagnosed most commonly at ages of 65 to 74 years. In the United States, an estimated 34,920 new cases of MM will be diagnosed in 2021, with an estimated 12,410 deaths, accounting for 1.8% of all new cancers and 2.0% of all cancer deaths.^(^ ^3^ ^)^ The estimated overall 5‐year survival is 55.6%.^(^ ^4^ ^)^

### Myeloma bone disease

Osteolytic lesions with or without diffuse osteopenia, pathologic fractures, and focal lytic lesions are common features seen in patients with MM. Myeloma bone disease (MBD) occurs in approximately 80% to 95% of patients.^(^ ^5^, ^6^ ^)^ MBD predominantly affects the axial skeleton and can have serious skeletal consequences such as spinal cord compression and pathologic fractures requiring radiotherapeutic and/or surgical intervention), commonly referred to as skeletal‐related events (SREs).^(^ ^7^ ^)^ Fractures are observed in approximately 50% of MM patients.^(^ ^6^ ^)^ Even in patients in remission or with low‐grade stable disease after stem cell transplantation, fractures were reported in up to 13% of patients.^(^ ^8^ ^)^ MBD can have debilitating effects on the quality of life of MM patients and in their survivorship with respect to severe pain, psychological distress, and loss of autonomy.^(^ ^9^ ^)^ Most importantly, it is also associated with increased morbidity and mortality.^(^ ^10^, ^11^, ^12^ ^)^

## Pathogenesis of MBD

Skeletal homeostasis is a complex and multifactorial process of interactions between the bone matrix, osteoclasts, osteoblasts, osteocytes, and the immune system.^(^ ^13^, ^14^ ^)^ Osteoclasts and osteoblasts are derived from distinct cellular lineages. Osteoclasts are derived from fusion of mononuclear cells of the monocyte–macrophage lineage.^(^ ^15^ ^)^ They are regulated by receptor activator of NF‐κB (RANK), its ligand RANKL, and the decoy receptor osteoprotegerin (OPG).^(^ ^16^ ^)^ Osteoblasts evolve from mesenchymal cells to osteocytes through a differentiation process called osteoblastogenesis. This process is regulated by the Wingless‐type (Wnt) signaling and β‐catenin pathways.^(^ ^17^, ^18^ ^)^ In normal bone metabolism, bone resorption and formation are regulated by a coupled function of osteoclast and osteoblast. Imbalance between bone‐resorbing osteoclasts and bone‐forming osteoblasts causes significant dysregulation of bone homeostasis and resultant MBD. The interactions among MM cells, residential cellular components of the bone, and immune cells favor the expansion of MM cells and the destruction of normal bone structures (Figure URE 1). It has also been shown that once the destructive bone disease occurs, it does not completely reverse even once MM is in remission. Therefore, developing new therapies targeting MBD is important not only for MM disease control, but also for the quality of life of MM survivors.

**Fig. 1:**
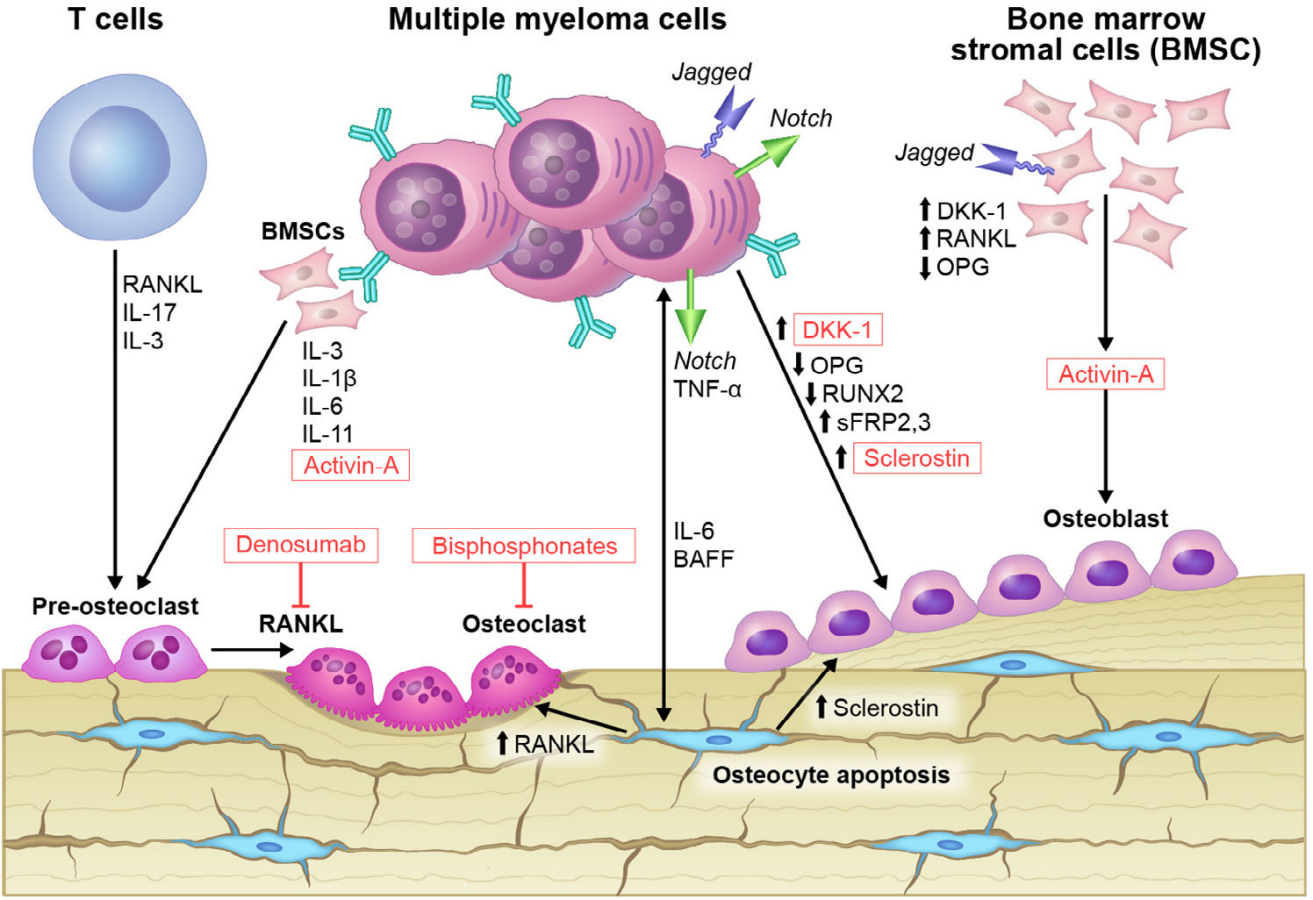
Simplified schematic overview of MBD. The interactions between MM cells and BMSCs together with T cells inside the bone favor cytokine production such as IL‐1β, IL‐6, IL‐11, IL‐3 and IL‐17. Such cytokines increase osteoclast activity and decrease osteoblastogenesis, leading to increased bone loss. The interaction of the aberrantly expressed Notch on MM cells with its Jagged ligand on adjacent MM cells or BMSCs induces increased production of RANKL and decrease OPG, favoring increased osteoclastogenesis. The interaction between MM cells and osteocytes is bidirectional. MM cell derived TN)‐α and Notch signaling initiates osteocyte apoptosis, which in turn increases MM cell proliferation, through signaling such as Notch and BAFF. Osteocyte apoptosis also increases RANKL and sclerostin, leading to bone absorption. Both MM cells and apoptotic osteocytes produces soluble factors such as sclerostin, DKK‐1, and the sFRPs, further suppresses osteoblastogenesis. MM cells also inhibit osteoblast differentiation by suppressing its critical transcriptional factor RUNX2. In addition, MM cells induce the secretion of activin‐A by BMSCs, which stimulates osteoclast growth and possibly inhibits osteoblast function. Abbreviations: BAFF, B‐cell activating factor; BMSC, bone marrow stromal cell; DKK, Dickkopf; IL, interleukin; MBD, myeloma bone disease; MM, multiple myeloma; OPG, osteoprotegerin; RANKL, receptor activator of NF‐κB ligand; RUNX2, Runt‐related transcription factor 2; sFRP, secreted Frizzled‐related protein; TNF, tumor necrosis factor.

### Upregulation of osteoclast activity

Increased activity of osteoclasts is observed in MM that occurs through several pathways: the RANK/RANKL pathway, the Notch signaling pathway, and other factors that favor osteoclastogenesis. RANKL is produced primarily by osteocytes and promotes osteoclast activity by binding to RANK. OPG is secreted by osteoblasts, bone marrow stromal cells (BMSCs), and osteocytes, and inhibits interaction of RANK with RANKL. OPG was shown to inhibit the development of osteolytic bone disease in MM.^(^ ^19^ ^)^ In general, an increase in the RANKL/OPG ratio favors bone destruction.^(^ ^20^, ^21^ ^)^ This is seen in inflammatory diseases such as rheumatoid arthritis and in several types of cancers. Direct interaction of MM cells with BMSCs leads to increased expression of RANKL, and decreased expression of OPG by BMSCs and osteocytes in the bone microenvironment. The activated intracellular Notch signaling pathway results in increased production of RANKL, which binds to RANK and promotes osteoclastogenesis.^(^ ^22^, ^23^, ^24^ ^)^ Interactions between BMSCs, MM cells, and immune cells induce release of proosteoclastogenic factors and several cytokines such as interleukin‐1b (IL‐1b), IL‐3, IL‐6, IL‐11, and IL‐17.^(^ ^24^ ^)^ These cytokines increase osteoclast activity and decrease osteoblastogenesis, leading to increased bone resorption. In addition, activin A, initially isolated as a gonadal protein, a member of the transforming growth factor‐β (TGF‐β) superfamily, is found to have a broad spectrum of biological functions including regulating the extracellular matrix formation and mineralization of the bone.^(^ ^25^, ^26^ ^)^ MM cells induce the secretion of activin A by BMSCs, which stimulates osteoclast growth and possibly inhibits osteoblast function.^(^ ^27^, ^28^ ^)^ Activin A levels are found elevated in MM patients, especially in those with advanced disease and extensive MBD.^(^ ^29^ ^)^

### Downregulation of osteoblast activity

Suppressed activity of osteoblasts occurs because of aberrant Wnt signaling in MM.^(^ ^30^ ^)^ Inhibitors of the canonical Wnt pathway, such as sclerostin, Dickkopf‐like protein 1 (DKK‐1), and soluble frizzled‐related proteins (sFRP), inhibit bone formation.^(^ ^31^, ^32^ ^)^ Sclerostin, a glycoprotein produced by osteocytes, impedes the activation of the canonical Wnt pathway, inhibiting osteoblast maturation and impairing bone mineralization.^(^ ^33^, ^34^, ^35^ ^)^ Furthermore, it induces apoptosis of osteoblasts through caspase activation and increases the RANKL/OPG ratio, resulting in enhanced osteoclastogenesis. Another antagonist of the Wnt pathway is DKK‐1, which is secreted by MM cells.^(^ ^36^ ^)^ Osteoblastogenesis and new bone formation are inhibited by the binding of DKK‐1 to lipoprotein receptor‐related protein (LRP)‐6. In addition, DKK‐1 enhances secretion of sclerostin^(^ ^37^ ^)^ and increases the RANKL/OPG ratio, resulting in increased osteoclastogenesis.^(^ ^38^ ^)^ Several other Wnt pathway regulatory factors including periostin, Runt‐related transcription factor 2, and Growth factor independence 1 are also deregulated in MM.^(^ ^24^ ^)^

As this brief discussion demonstrates, MBD is complex and involves the dysregulation of several pathways and physiologic processes. Detailed pathophysiologic mechanisms of MBD have been described in detail in other reports.^(^ ^24^, ^39^ ^)^ Furthermore, recent preclinical work has implicated the Hippo pathway in the pathogenesis of MBD.^(^ ^40^ ^)^ In general, the mechanisms involved are still being uncovered, and better understanding of the mechanisms involved in MBD will result in innovative treatment approaches.

### Osteocytes

Although the formation and resorption of the bone is directly attributed to osteoblasts and osteoclasts, the majority of cells in the bone microenvironment are osteocytes, which constitute >95% of bone cells. Osteocytes are the central regulators of both osteoblast and osteoclasts. Osteocytes secrete sclerostin, an inhibitor of bone formation, and RANKL, which promotes osteoclastogenesis. Apoptotic osteocytes (seen in disuse, glucocorticoid treatment, estrogen deficiency) induces osteoclast precursor recruitment and bone resorption.^(^ ^41^, ^42^ ^)^ MM cells interact with osteocytes in the bone microenvironment and can promote MBD by increasing osteocyte apoptosis, increase sclerostin and RANKL production, and inhibit osteoblast differentiation. The interactions of MM cells and osteocytes are reciprocal. Osteocytes are shown to activate Notch signaling via Notch3, which leads to increased MM cell proliferation, and has the capacity to change the Notch receptor repertoire expressed by MM cells.^(^ ^23^ ^)^ In vivo studies inhibiting Notch signaling, alone or in combination with other anti‐apoptotic treatment led to the inhibition of MM cell growth.^(^ ^43^, ^44^ ^)^

## Treatment of MBD

In patients with MM and/or MBD, various treatment strategies are available. Management of underlying MM is crucial as MBD will ensue or progress without adequate control of underlying MM. Preventative therapies are needed to delay MBD progression.^(^ ^45^ ^)^ Currently, the mainstay of preventative therapies are antiresorptive agents (Table 1).^(^ ^46^, ^47^, ^48^, ^49^, ^50^, ^51^, ^52^, ^53^, ^54^, ^55^ ^)^ However, these agents are limited in not being able to promote new bone formation or repair existing bone lesions.^(^ ^56^ ^)^ Newer anabolic agents that promote osteoblastogenesis and bone formation can potentially repair existing bone lesions and could improve MBD (Table 2).^(^ ^57^, ^58^, ^59^, ^60^, ^61^, ^62^, ^63^ ^)^ In addition to bone‐modifying agents, antitumor therapies, radiotherapy, and surgery are other options used in the treatment of MBD.^(^ ^11^, ^45^ ^)^

**Table 1:** Phase 3 studies evaluating bone‐targeting agents in MM

Study	Phase	Patient population	Intervention	Results
Lahtinen et al.^(^ ^48^ ^)^ (1992)	3	Newly diagnosed MM	Clodronate oral 2400 mg daily (*n* = 168) versus placebo (*n* = 168) × 24 months.	Progression of osteolytic lesions: 24% (clodronate) versus 12% (placebo), *p* = 0.03.
Berenson et al.^(^ ^49^ ^)^ (1996)	3	Durie‐Salmon Stage III MM with ≥1 lytic bone lesion	Pamidronate IV 90 mg every 4 weeks (*n* = 196) versus placebo (*n* = 181) × 9 months.	SRE after 9 cycles: pamidronate 24% versus placebo 41%, *p* = 0.001. No OS difference.
Berenson et al.^(^ ^50^ ^)^ (1998)	3	Durie‐Salmon Stage III MM with ≥1 lytic bone lesion	Pamidronate IV 90 mg every 4 weeks (*n* = 198) versus placebo (*n* = 179) × 21 months.	SRE after 21 cycles: pamidronate 50% versus placebo 58%, *p* = 0.02. No OS difference.
McCloskey et al.^(^ ^51^, ^52^ ^)^ (1998, 2001)	3	MM with 2 of 3 of the following: (1) clonal bone marrow plasmacytosis; (2) blood or urine monoclonal protein; (3) lytic bone lesions	Clodronate oral 1600 mg daily (*n* = 264) versus placebo (*n* = 272) until SRE or hypercalcemia.	Nonvertebral fractures: 6.8% (clodronate) versus 13.2% (placebo), *p* = 0.04. Vertebral fractures: 38% (clodronate) versus 55% (placebo), *p* = 0.01. No OS difference.
Gimsing et al.^(^ ^53^ ^)^ (2010)	3	Newly diagnosed MM	Pamidronate IV 30 mg monthly (*n* = 250) versus pamidronate IV 90 mg monthly (*n* = 252) × 3 years.	Median time to first SRE: 9.2 months (pamidronate 90 mg) versus 10.2 months (pamidronate 30 mg), *p* = 0.6. No OS difference.
Morgan et al.^(^ ^54^, ^55^ ^)^ (2010, 2011)	3	Newly diagnosed MM	Zoledronic acid IV 4 mg every 3–4 weeks (*n* = 981) versus clodronic acid oral 1600 mg daily (*n* = 979) until disease progression.	Skeletal related events: 27% (zoledronic acid) versus 35% (clodronic acid), *p* = 0.0004. Median OS: 50.0 months (zoledronic acid) versus 44.5 months (clodronic acid), *p* = 0.04.
Himelstein et al.^(^ ^46^ ^)^ (2017)	3	MM with ≥1 lytic bone lesion	Zoledronic acid IV every 4 weeks (*n* = 139, MM subgroup) versus every 12 weeks (*n* = 139, MM subgroup) × 2 years.	Non‐inferior difference in probability of SRE with between‐group difference, 0.06 (99.9% CI, −0.12 to 0.24); *p* = 0.14.
Raje et al.^(^ ^47^ ^)^ (2018)	3	Newly diagnosed MM with ≥1 lytic bone lesion	Denosumab SC + placebo IV (*n* = 859) versus zoledronic acid IV + placebo SC (*n* = 859).	Median time to first SRE: 22.8 months (denosumab) versus 24.0 months (zoledronic acid), *p* = 0.01 for non‐inferiority of denosumab.

Abbreviations: IV, intravenous; MM, multiple myeloma; OS, overall survival; SC, subcutaneous; SRE, skeletal related event.

**Table 2:** Clinical studies evaluating novel treatment approaches to MBD

Study	Phase	Target	Drug	Intervention	Notable findings
Abdulkadyrov et al.^(^ ^57^ ^)^ (2014)	2a	Activin A	Sotatercept (recombinant activin receptor type IIA ligand trap)	4:1 randomization. Sotatercept SC (0.1, 0.3, or 0.5 mg/kg) or placebo every 28 days × 4 cycles + melphalan, prednisone, thalidomide (*n* = 30).	Anabolic effect with sotatercept in patients who had not received bisphosphonates within 2 months prior to study initiation. Increased hemoglobin levels with sotatercept therapy.
NCT01562405^(^ ^60^ ^)^	1	Activin A	Sotatercept	Sotatercept + lenalidomide or pomalidomide + dexamethasone in RRMM	Pending.
Iyer et al.^(^ ^59^ ^)^ (2014)	1b	DKK‐1	BHQ880 (IgG1 anti‐DKK‐1 monoclonal antibody)	BHQ880 IV (3–40 mg/kg) + zoledronic acid IV (4 mg) intravenously every 28 days × 24 cycles (*n* = 28).	No DLTs. RP2D BHQ880 10 mg/kg, mainly based on target saturation data. General trend toward increased BMD observed over time.
NCT01302886^(^ ^61^ ^)^	2	DKK‐1	BHQ880	BHQ880 in high‐risk smoldering myeloma	Pending.
NCT01337752^(^ ^62^ ^)^	2	DKK‐1	BHQ880	BHQ880 or placebo + bortezomib, dexamethasone in NDMM who are not candidates for bisphosphonate therapy.	Pending.
NCT01457417^(^ ^63^ ^)^	1	DKK‐1	DKN‐01 (IgG4 anti‐DKK‐1 monoclonal antibody)	DKN‐01 in MM.	Pending.
Diamond et al.^(^ ^58^ ^)^ (2020)	2a	PTH	Teriparatide (recombinant PTH)	SC teriparatide 20 μg daily × 12 months (*n* = 12).	Serum P1NP increased by 4.8‐fold (0.5–13) and lumbar spine QCT BMD by 43.8% (12.2%–100%). No new fractures or lytic lesions were recorded. Increase in serum P1NP concentrations from 33 to 151 μg/L (*p* <0.001) from baseline after 12 months. Lumbar spine BMD increased 43.8% from baseline (*p* < 0.001).

Abbreviations: BMD = bone mineral density; DKK‐1 = Dickkopf‐1; DLT = dose‐limiting toxicity; IV = intravenous; MBD = myeloma bone disease; MM = multiple myeloma; N = number; NDMM = newly diagnosed multiple myeloma; P1NP = pro‐peptide of type 1 procollagen; PTH = parathyroid hormone; QCT, quantitative computed tomography; RP2D = recommend phase 2 dose; RRMM = relapsed refractory multiple myeloma; SC, subcutaneous.

### Antiresorptive therapies

#### Bisphosphonates

Bisphosphonates (BPs) have been the most widely used antiresorptive medication in treating MM and MBD. BPs are pyrophosphate analogues that avidly bind to hydroxyapatite and get incorporated into the bone matrix.^(^ ^64^ ^)^ All BPs have two phosphate groups with a central carbon atom; however, their affinity for binding with hydroxyapatite depends on the composition of the side chains.^(^ ^65^ ^)^ Bisphosphonates are classified into two main types based on their affinity for binding with hydroxyapatite: (i) BPs containing nitrogen, such as pamidronate and zoledronic acid, and (ii) non–nitrogen‐containing BPs, such as etidronate and clodronate; the nitrogen‐containing BPs are 100‐fold to 10,000‐fold more potent.^(^ ^66^ ^)^ Bisphosphonates suppress osteoclast activity and thus favorably change the balance between bone formation and destruction, resulting in increased bone mass, and there is experimental evidence suggesting that BPs may have a mitogenic effect on osteoblasts. One of proposed pathway is BP's effect on suppressing RANKL and increasing OPG in human osteoblasts, leading to bone formation.^(^ ^67^, ^68^, ^69^, ^70^ ^)^

In terms of SREs, none of the BPs approved by the US Food and Drug Administration (FDA) (clodronate, pamidronate, or zoledronic acid) showed superiority over the others in a recently conducted Cochrane network meta‐analysis.^(^ ^70^ ^)^ However, in a randomized controlled study, zoledronic acid was shown to be superior to clodronate for decreasing SREs in symptomatic newly diagnosed patients with MM.^(^ ^56^ ^)^ In addition, patients randomized to the zoledronic acid arm showed improved overall survival and progression‐free survival, in addition to that attributed to the preventative effects of SREs. This increase in survival is in keeping with findings from preclinical studies and can be attributable to direct or indirect anti‐MM effects.^(^ ^71^ ^)^

Adverse effects of BPs include acute‐phase reactions, which present within the first few hours or days after initiation of BP and often resolve with symptomatic management.^(^ ^72^, ^73^ ^)^ Other serious adverse events may occur, including renal impairment, osteonecrosis of jaw (ONJ), and atypical femoral fractures. Renal impairment can occur with BPs because the kidneys eliminate BP exclusively.^(^ ^64^, ^65^ ^)^ Renal damage is multifactorial,^(^ ^74^ ^)^ and higher risk of renal damage is seen in high doses of BP and with faster infusion rates.^(^ ^75^ ^)^ The true incidence of renal damage following BP therapy is unknown. A study evaluating the use of BP in patients with breast cancer and MM found renal damage in 10.7% of patients who received zoledronic acid and 9.3% of patients who received pamidronate.^(^ ^75^ ^)^ Renal damage from BPs is a notable adverse event because renal damage can progress to renal failure.^(^ ^72^ ^)^ Another serious adverse event following BP therapy is ONJ, which occurs in a minority of patients. The risk of ONJ is dependent on the dose and duration of exposure to BP.^(^ ^76^ ^)^ Other risk factors for the development of ONJ following BP therapy include dental infections, advanced age, smoking, diabetes mellitus, and therapy with cyclophosphamides.^(^ ^77^, ^78^, ^79^ ^)^ In the event of BP‐induced ONJ, the BP is discontinued, and most patients can be managed with conservative measures such as optimal dental hygiene and antibiotics.^(^ ^80^ ^)^ Some patients may require surgical excision of necrosed bone. Atypical femoral fractures following BP therapy have been recognized as a serious consequence of prolonged exposure to BPs.^(^ ^81^, ^82^, ^83^ ^)^ Patients may or may not present with pain in the thigh or groin region, and potential atypical fractures should be investigated in patients on BPs who present with skeletal pain.^(^ ^84^ ^)^ The pathogenesis of atypical fractures following BP therapy has been postulated to be related to long‐term suppression of bone remodeling; however, the exact mechanisms remain unknown.^(^ ^72^, ^85^ ^)^

#### Denosumab

Excessive production of a key component, RANKL, in the pathogenesis of MBD has been associated with increased bone resorption.^(^ ^84^ ^)^ Denosumab is a fully human monoclonal antibody against RANKL that impedes the interaction of RANKL with RANK. Initial phase I/II clinical trials of denosumab demonstrated decreased bone resorption markers, resulting in a decrease in bone resorption.^(^ ^86^, ^87^ ^)^

To further determine the role of denosumab in the management of MBD in patients with MM, a phase III randomized, placebo‐controlled study of 1718 patients with newly diagnosed MM with at least one documented lytic bone lesion was conducted.^(^ ^47^ ^)^ Patients were randomized in a 1:1 allocation ratio to receive 120 mg denosumab subcutaneously with intravenous placebo or to receive 4 mg intravenous zoledronic acid with subcutaneous placebo, every 4 weeks. Patients in the denosumab group had a similar time to first SRE (hazard ratio [HR] 0.98; 95% confidence interval [CI], 0.85–1.14) as patients in the zoledronic acid group. Further post hoc analysis using a 15‐month landmark timepoint revealed that denosumab was superior to zoledronic acid with respect to the time to first SRE development (HR 0.66; 95% CI, 0.44–0.98). No difference in overall survival was observed between the groups. Further subgroup analysis to evaluate the effect of denosumab on progression‐free survival showed that denosumab had a statistically significant greater progression‐free survival rate when compared to zoledronic acid, suggesting an additive effect of denosumab with antimyeloma therapy.^(^ ^88^ ^)^ Patients receiving denosumab had fewer adverse events related to renal toxicity (10% vs. 17%), likely because the clearance of denosumab, in contrast to BPs, occurs via the reticuloendothelial system and is independent of renal function.^(^ ^90^, ^91^ ^)^ Moreover, acute‐phase reactions, a classic adverse event that occur frequently in patients who receive BPs, occurred in 5% of patients receiving denosumab, compared to 9% of patients receiving zoledronic acid.^(^ ^91^ ^)^ In contrast, the incidence of hypocalcemia was higher with denosumab (17%) compared to zoledronic acid (12%). The incidence rate of ONJ in patients receiving denosumab was similar to that of patients receiving zoledronic acid (4% vs. 3%, *p* = 0.15). This study confirmed that denosumab as an option as a bone‐modifying agent in MM and may be particularly useful in MM patients with renal insufficiency that precludes the use of zoledronic acid.

Denosumab should not be abruptly discontinued due to the risk of rebound osteoporosis,^(^ ^92^ ^)^ and multiple spontaneous vertebral fractures have been observed in studies with longer follow‐up after stopping denosumumab.^(^ ^93^ ^)^ MM patients who start denosumuab should be counseled that that they must continue denosumab at minimum every 6 months or bridge to BP therapy if they choose to discontinue every 4‐week denosumab dosing at some point to prevent rapid bone turnover.^(^ ^94^ ^)^ If adherence to denosumab therapy is anticipated to be a challenge, then BP therapy may be preferred in such patients. Monitoring bone resorption biomarkers such as C‐telopeptide (CTX) or N‐telopeptide (NTX) to determine the optimal timing of BP bridging after denosumab discontinuation has also been explored although has not been clearly defined for routine practice.

### Anabolic therapies

#### Anti‐sclerostin antibodies

MBD is a consequence of both an increase in bone resorption and a decrease in bone formation.^(^ ^95^ ^)^ Although BPs can reduce the rate of bone resorption, they do not affect bone formation, and thus MBD is not completely preventable with the use of BPs alone. Encoded by the *SOST* gene, sclerostin is produced by osteocytes, binds to Wnt co‐receptors, and antagonizes the pathway.^(^ ^34^ ^)^ This is an important pathway in the pathogenesis of osteoporosis; however, its role in preventing or treating MBD has not yet been well established. In an in vitro study, MM cells co‐cultured with osteocytes led to increased expression of SOST/sclerostin in osteocytes, decreased Wnt signaling/β‐catenin, and decreased osteoblast differentiation.^(^ ^23^ ^)^

A study in mice with MM showed similar findings with raised levels of sclerostin and a 50% decrease in OPG, which correlated with a decrease in osteoblast markers.^(^ ^23^ ^)^ Other studies showed that anti‐sclerostin treatment in mice with MM increased trabecular bone volume and thickness.^(^ ^97^, ^98^ ^)^ In a study of patients with MM, elevated sclerostin levels were found in those with abnormal bone remodeling.^(^ ^98^ ^)^

Recent trials have tested humanized anti‐sclerostin monoclonal antibodies romosozumab and blosozumab in patients with osteoporosis. A phase I randomized and controlled trial of subcutaneous or intravenous romosozumab versus placebo in healthy men and postmenopausal women revealed that patients who received romosozumab showed increased serum levels of bone formation markers and decreased serum levels of a bone resorption marker in comparison to patients who received placebo.^(^ ^99^ ^)^ In a phase II, multicenter, parallel‐group study, postmenopausal women with low bone mass who received romosozumab had increased bone density and bone formation, with decreased bone resorption, compared with women who did not receive romosozumab.^(^ ^100^ ^)^ An international, randomized, double‐blind, parallel‐group phase III trial (Fracture Study in Postmenopausal Women with Osteoporosis [FRAME]) of romosozumab at a dose of 210 mg once monthly showed a lower risk of vertebral fracture at 12 months in the patients receiving romosozumab compared to placebo.^(^ ^101^ ^)^

Romosozumab is generally well tolerated. In the large phase III FRAME trial, injection site reactions were seen in 5.2% of patients in the romosozumab group, compared to 2.9% in the placebo group.^(^ ^101^ ^)^ The frequencies of mortality and cardiac disorders were similar between the groups. ONJ was detected in two patients with recognized risk factors in the romosozumab group. An atypical femoral fracture occurred in one patient 3.5 months after the first dose of romosozumab. Romosozumab was approved in 2019 in Japan and the United States for the treatment of osteoporosis in patients at high risk of fracture. The efficacy of antisclerostin antibodies has not been evaluated in patients with MM.

McDonald et al.^(^ ^97^ ^)^ evaluated the effect of antisclerostin antibody alone or in combination with BPs in myeloma murine models. Results showed that antisclerostin antibody therapy prevented suppression of osteoblastic bone formation which is induced by myeloma, prevented bone loss, lowered the number of osteolytic lesions, and most importantly, increased bone strength and fracture resistance. Combination treatment with an antisclerostin antibody and zoledronic acid improved bone mass, strength, and fracture resistance when compared to treatment with zoledronic acid monotherapy. Thus, antisclerostin antibodies alone, or in combination with other therapies may also be a promising therapeutic approach for future investigation in MM.

#### Parathyroid hormone

In the osteoporotic setting, parathyroid hormone (PTH) has been shown to have anabolic effects; however, the exact mechanisms for the anabolic effect remain unclear.^(^ ^102^ ^)^ It has been postulated that PTH may increase osteoblastogenesis as well as inhibit sclerostin, a potent promoter of osteoclastogenesis. A recombinant form of PTH, teriparatide, and a recombinant analog of PTH‐related peptide, abaloparatide, are FDA‐approved for women with osteoporosis.^(^ ^104^, ^105^ ^)^ Several preclinical studies have evaluated the effects of PTH administration in MM. For example in mouse models, PTH treatment has been shown to increase bone mineral density (BMD) via upregulation of osteoblasts, and gene expression profiling of whole myeloma bones demonstrated increased expression of osteoblastic markers and reduced expression of osteoclastic markers with PTH exposure.^(^ ^105^ ^)^ Importantly, myeloma cells did not express PTH receptors, and PTH did not impact myeloma cell growth in vitro.^(^ ^105^ ^)^ Several case reports have shown teriparatide to improve BP‐associated ONJ by showing significant healing of necrotic bone.^(^ ^106^ ^)^

Concerns about the safety of PTH use the MM patients remain, particularly regarding the mitogenic potential of anabolic agents such as PTH analogues in promoting MM growth. For example, high levels of PTH may enhance the growth of MM cells via the secretion of IL‐6.^(^ ^107^ ^)^ In prostate cancer, a higher serum level of PTH has been associated with an increase in skeletal metastasis.^(^ ^108^ ^)^ As such, in the label, teriparatide is contraindicated in patients with a history of osteosarcoma, or with increased risk of osteosarcoma with prior radiation to the bone, or metastatic bone disease.^(^ ^109^ ^)^ Its use could also potentially exacerbate hypercalcemia that can occur in MM patients. Recently, a small pilot study of the use of teriparatide in MM was reported in which 12 patients were treated with subcutaneous teriparatide 20 μg daily without concurrent BP use for 12 months.^(^ ^58^ ^)^ Overall, teriparitide was well tolerated, and no new SREs or hypercalcemia was observed while patients were on study. Importantly, teriparatide did result in an increase in BMD when measured in the lumbar spine by 43.8% from baseline (*p* < 0.001). Thus, the encouraging results from this study could suggest a role of anabolic agents in treatment of MBD, and the utility of PTH in the treatment of MBD warrants further investigation.

#### Anti‐DKK‐1

DKK‐1 is a potent regulator of the Wnt signaling pathway and is found to be elevated in MM. It inhibits the Frizzled co‐receptor LRP6 and is produced by BMSCs as well as malignant plasma cells. In the presence of sclerostin, DKK‐1 decreases β‐catenin, which reduces differentiation of osteoblasts.^(^ ^37^, ^111^ ^)^ A humanized immunoglobulin G (IgG) anti–DKK‐1 monoclonal antibody, BHQ880, has been evaluated in vitro and in vivo.^(^ ^111^ ^)^ BHQ880 was successful at reversing the inhibiting effect of DKK‐1 on osteoblast differentiation and promoted bone formation in a murine model of human MM. BHQ880 also inhibits MM cell growth and its negative effect on osteoblastogenesis, and reduced IL‐6 secretion. No direct effects were detected on osteoclastogenesis. Clinically, the use of BHQ880 has been evaluated in combination with zoledronic acid in a phase Ib study with 28 patients, and there was a trend toward increased BMD with treatment over time.^(^ ^59^ ^)^ However, because concurrent zoledronic acid was administered in this study, the relative impact of BHQ880 on bone remodeling was uncertain in this study. A phase II study of BHQ880 in high‐risk smoldering MM (NCT01302886)^(^ ^61^ ^)^ and a randomized placebo‐controlled phase II study of BHQ880 in untreated MM patients who are not candidates for BP therapy (NCT01337752)^(^ ^62^ ^)^ are ongoing which will further define the role of anti‐DKK‐1 treatment in the setting of MBD.

#### Other therapies with undetermined role in MBD

TGF‐β has been implicated to play a role in MBD.^(^ ^45^ ^)^ The use of a TGF‐β inhibitor, neutralizing antibody 1D11, in mice showed increased osteoblast differentiation and improved bone disease, yet no improvement in overall tumor burden was noted.^(^ ^112^ ^)^ Further evaluation is needed because TGF‐β can act as both a tumor suppressor and an oncogene, and the long‐term side effects of using TGF‐β–neutralizing antibodies have not been evaluated. Another agent under investigation is sotatercept, a soluble recombinant activin receptor type 2A ligand fused to the human immunoglobulin G (IgG) Fc domain, which disrupts downstream cascades by binding to activin A/B plus members of the TGF‐β family. In a phase II trial in newly diagnosed and/or relapsed MM patients, the addition of sotatercept to melphalan, prednisolone, and thalidomide revealed increased levels of bone‐specific alkaline phosphatase, a biomarker for bone formation.^(^ ^57^ ^)^ Other antitumor therapies with effects on bone metabolism have also been evaluated but are beyond the scope of this review.

## Society Guidelines

Several societies have developed guidelines for the screening, prevention, monitoring, and treatment of MBD in patients with MM. Here we highlight some of the most clinically relevant recommendations.

### Screening

The National Comprehensive Cancer Network (NCCN) recommends imaging of any patient with suspected MM.^(^ ^113^ ^)^ For initial diagnostic workup, they recommend the use of whole‐body low‐dose computed tomography (CT) or FDG‐PET/CT. When advanced imaging is not available, a skeletal survey is acceptable; however, it is significantly less sensitive. Following treatment of MM, the NCCN panel recommends use of advanced imaging, including whole‐body fluorodeoxyglucose (FDG)‐positron emission tomography (PET)/CT, low‐dose CT scan, or whole‐body magnetic resonance imaging (MRI) without contrast, as clinically indicated for follow‐up. The NCCN also recommend using the same imaging modality as used for the initial assessment.

### Prevention and treatment of MBD

Several guidelines from international organizations, including the American Society of Clinical Oncology (ASCO),^(^ ^114^ ^)^ British Committee for Standards in Haematology,^(^ ^115^ ^)^ European Myeloma Network,^(^ ^116^ ^)^ European Society for Medical Oncology,^(^ ^1^ ^)^ International Myeloma Working Group (IMWG),^(^ ^94^ ^)^ and NCCN^(^ ^117^ ^)^ recommend initiating BP therapy in all MM patients who require systemic chemotherapy regardless of presence of underlying bone disease. Here we focus on the ASCO and IMWG recommendations.^(^ ^95^, ^115^ ^)^ They recommend initiating BP therapy in active myeloma requiring systemic chemotherapy with or without lytic bone lesions or compression fractures seen on imaging. The use of BPs is not recommended in patients with solitary plasmacytoma, smoldering (asymptomatic) MM, and in patients with monoclonal gammopathy of undetermined significance unless they have osteopenia or osteoporosis.

The ASCO guidelines recommend the use of pamidronate (90 mg administered over a minimum of 2 h) or zoledronic acid (4 mg administered over a minimum of 15 min) every 3 to 4 weeks. Alternatively, they recommend using denosumab given its non‐inferior efficacy when compared to zoledronic acid in a large phase III study,^(^ ^47^ ^)^ in the section of the Treatment of BMD, Table 1. The IMWG preferred options include zoledronic acid (with or without MBD present on imaging) and denosumab (only when MBD present on imaging) and also should be considered for patients with renal impairment. A second option would be pamidronate when zoledronic acid or denosumab are not available or contraindicated.

In patients with mild to moderate renal impairment (defined as an estimated creatinine clearance between 30 and 60 ml/min), a reduced dosage of zoledronic acid with no changes in infusion time or interval is recommended. Zoledronic acid is not recommended in patients with severe renal impairment. In patients with existing severe renal impairment (serum creatinine level greater than 3.0 mg/dl (265 mmol/L) or an estimated creatinine clearance of <30 ml/min), pamidronate (90 mg administered over 4 to 6 h), or denosumab are recommended.

The ASCO guidelines recommend treatment with bone‐modifying agents for a period of up to 2 years. In patients in whom BPs are withdrawn, the BPs should be resumed upon new onset of SREs. Less frequent dosing (every 3 months rather than every 3 to 4 weeks) has been evaluated^(^ ^46^, ^118^ ^)^ and should be considered in patients with stable or responsive disease. The IMWG recommends zoledronic acid monthly during initial therapy and in patients with less than a very good partial response (VGPR) as per IMWG response criteria. However, if patients achieve at least a VGPR after receiving monthly administration for ≥12 months, then a decreased frequency of every 3–6 months, or yearly based on osteoporosis dosing, or stopping zoledronic acid can be considered. If discontinued, zoledronic acid should be reinitiated at the time of biochemical relapse. If denosumab is used, it should be administered monthly and should not be discontinued abruptly given its reversible mechanism of action and risk of rebound osteoporosis. Data on the optimal approach to discontinue denosumab is lacking, and current recommendations are to either administer a single dose of zoledronic acid at least 6 months after the last dose of denosumab or continue denosumab every 6 months after discontinuation of the monthly injection.

Regular monitoring of patients receiving BPs is needed. Serum creatinine should be evaluated prior to each dose of BP therapy, serum calcium should be monitored regularly, and vitamin D should be monitored intermittently. Every 3 to 6 months, patients should be evaluated for the presence of albuminuria in a spot urine sample, and if albuminuria is detected, the BP should be discontinued, and a 24‐h urine collection should be obtained. The use of biochemical bone metabolism markers to monitor the effects of bone‐modifying agents has not been well studied and there is no formal recommendation at this time. Finally, prior to initiation of BPs, all patients should have a comprehensive dental exam and be advised to maintain excellent oral hygiene and to avoid dental extractions while on BP therapy.

## Conclusion

The overall survival of MM patients has improved significantly over the last two decades with the incorporation of new drugs to the MM therapeutic armamentarium. However, MBD is a common complication of MM that significantly contributes to patient morbidity and mortality. The mainstay of treatment of MBD has been with antiresorptive agents including BPs and denosumab, which have been proven to be efficacious. However, these therapies are not without adverse events, which need to be recognized and treated appropriately. Newer agents for the management of MBD are under development and could potentially change the treatment landscape in patients with MM and MBD. A higher fracture rate above the general population is observed in MM patients, even those who achieve and maintain deep remissions after systemic chemotherapy. Moreover, with longer durations of response and overall survival as well as greater exposure to corticosteroids as part of myeloma therapy, osteoporotic insufficiency fractures relative to pathologic fractures from new lytic lesions will likely becoming increasingly relevant for patients in long‐term survivorship. Future directions in managing MBD include (i) targeting specific MM patient populations for aggressive MBD therapy who are at high‐risk for progressive MBD such as genetically defined high‐risk MM patients; (ii) potentially validating the use of bone‐turnover markers in larger studies to optimize the use of MBD therapy in MM patients; and (iii) exploring the use of novel osteoporosis agents such as the anti‐sclerostin monoclonal antibody romosozumab in MBD.

## Disclosures

Huifang Lu declares research funding from Genentech. Xerxes Pundole: None; now working at Amgen Inc.; all related research contributions were completed while employed at The University of Texas MD Anderson Cancer Center. Hans C. Lee declares consulting fees from Amgen, Celgene, Genentech, GlaxoKlineSmith, Janssen, Sanofi, and Takeda and research funding from Amgen, Celgene, Daiichi Sankyo, GlaxoKlineSmith, Janssen, Regeneron, and Takeda.

## Author Contributions

Huifang Lu: Synthesis of information, literature search, manuscript writing and editing. The author takes full responsibility for the integrity of the data analysis. Xerxes Pundole: Synthesis of information, literature search, manuscript writing and editing. Hans C. Lee: Synthesis of information, literature search, manuscript writing and editing.

### Peer Review

The peer review history for this article is available at https://publons.com/publon/10.1002/jbm4.10518.
